# Exercise-Induced Desaturation in Patent Foramen Ovale: Mechanisms, Diagnostic Approach, and Resolution After Closure—A Narrative Review with an Illustrative Case

**DOI:** 10.3390/jcm15041523

**Published:** 2026-02-14

**Authors:** Martina Podolec, Jiří Dostál, Petr Volf, Aneta Dvořáková, Martin Mates

**Affiliations:** 1 Department of Cardiology, 1st Medical Faculty of Charles University and Na Homolce Hospital, Roentgenova 37/2, 150 30 Prague, Czech Republic; petr.volf@fnmh.cz (P.V.); aneta.dvorakova@fnmh.cz (A.D.); martin.mates@fnmh.cz (M.M.); 2 1st Department of Internal Medicine—Cardioangiology, Faculty of Medicine in Hradec Králové, Charles University, Šimkova 870, 500 03 Hradec Králové, Czech Republic; dostal@centrumsportmed.cz; 3 Department of Internal Medicine, 2nd Medical Faculty of Charles University, V Úvalu 84, 150 06 Prague, Czech Republic

**Keywords:** patent foramen ovale (PFO), exercise-induced desaturation (EID), right-to-left (R–L) shunt, cardiopulmonary exercise testing (CPET), exertional dyspnoea

## Abstract

Exercise-induced desaturation is an uncommon but clinically significant manifestation of patent foramen ovale, which is present in approximately one-quarter of the general population. Although patent foramen ovale is usually asymptomatic, exertion may provoke transient right-to-left shunting when dynamic changes in venous return and intrathoracic pressure favour intermittent right-to-left transit across the interatrial septum. This narrative review synthesises current evidence on exertion-provoked shunting and its contribution to otherwise unexplained dyspnoea and hypoxaemia. To illustrate these concepts, we present an illustrative case with marked exercise-induced desaturation in the absence of pulmonary disease. The evaluation combined contrast transthoracic and transoesophageal echocardiography with cardiopulmonary exercise testing, and the shunt magnitude was quantified invasively using catheter-based thermodilution at rest and during Valsalva provocation. Six months after percutaneous closure, repeat cardiopulmonary exercise testing showed complete resolution of exercise-induced desaturation without a statistically significant change in exercise tolerance (work performed). Notably, normalisation of oxygen saturation during exercise may occur without a measurable increase in maximal exercise capacity. Overall, patent foramen ovale-mediated right-to-left shunting is an under-recognised yet potentially reversible cause of exertional hypoxaemia; diagnosis typically requires deliberate physiological provocation and integrated imaging, and closure can be considered in carefully selected individuals.

## 1. Introduction

The patent foramen ovale (PFO) is a remnant of the foetal circulation that results from the incomplete fusion of the septum primum and septum secundum. It is an exceedingly common anatomical finding, present in nearly one out of every four people; for the vast majority of these individuals, it carries no clinical implications. However, in a subset of the population, the PFO can become a conduit for significant pathology, serving as a pathway for clinically important right-to-left (R–L) shunting and embolic events. It is most frequently associated with cryptogenic stroke via paradoxical embolism, migraine with aura, or specific clinical entities such as decompression illness and platypnoea–orthodeoxia syndrome (POS) [1,2].

A less frequently recognised manifestation of PFO is the desaturation of arterial blood during physical exertion, driven by a transient, exercise-induced R–L shunt. This phenomenon, termed ‘exercise-induced desaturation’ (EID), most likely results from a temporary elevation in right atrial (RA) pressure and/or altered RA flow dynamics that functionally override the usual interatrial pressure gradient. Importantly, clinical evidence has demonstrated that percutaneous PFO closure can effectively ameliorate hypoxaemia and alleviate exertional dyspnoea in carefully selected patients [3,4]. Notably, experimental work in healthy adults suggests that an asymptomatic PFO is typically associated with only small, intermittent atrial-level shunting and does not, by itself, explain the inter-individual variability in gas exchange impairment during maximal exercise, supporting the concept that clinically relevant EID requires additional anatomical and/or functional triggers [5].

The mechanism underlying EID shares conceptual similarities with POS, yet is physiologically distinct. This conceptual framework aligns with two critical clinical observations: first, that PFO-mediated hypoxaemia often represents an inducible, transient event rather than a fixed or permanent shunt, and second, that such desaturations remain a rare clinical phenomenon despite the high (~25%) prevalence of PFO in the general population [6,7].

Despite its clinical significance, EID remains a sparsely documented manifestation of PFO and lacks a fully standardised nomenclature. Nonetheless, emerging evidence indicates that percutaneous PFO closure can effectively resolve arterial hypoxaemia and may substantially improve functional capacity [2,3,8,9]. This work reviews the current literature and presents an illustrative clinical case employing rigorous physiological assessments—including cardiopulmonary exercise testing (CPET) and invasive catheter-based thermodilution—to quantify the R–L shunt and evaluate the therapeutic efficacy of percutaneous intervention.

This article was conceived as a narrative review to integrate physiological, diagnostic, and interventional evidence relating to PFO-mediated exertional hypoxaemia. We identified the relevant literature by searching major biomedical databases (PubMed/MEDLINE, Scopus, Web of Science, and Google Scholar) from inception to December 2025, using combinations of terms including “patent foramen ovale”, “right-to-left shunt”, “exercise-induced desaturation”, “exercise-induced hypoxaemia”, “provoked desaturation”, “platypnoea–orthodeoxia”, “contrast echocardiography”, and “cardiopulmonary exercise testing”. We additionally screened the reference lists of key papers and reviews to capture relevant case reports/series and physiological studies. Given the rarity of this phenotype, we prioritised clinically informative observational cohorts, case series, and mechanistic reports.

## 2. Platypnoea–Orthodeoxia Syndrome (POS) vs. Exercise-Induced Desaturation (EID) in Patients with PFO

**Figure 1:**
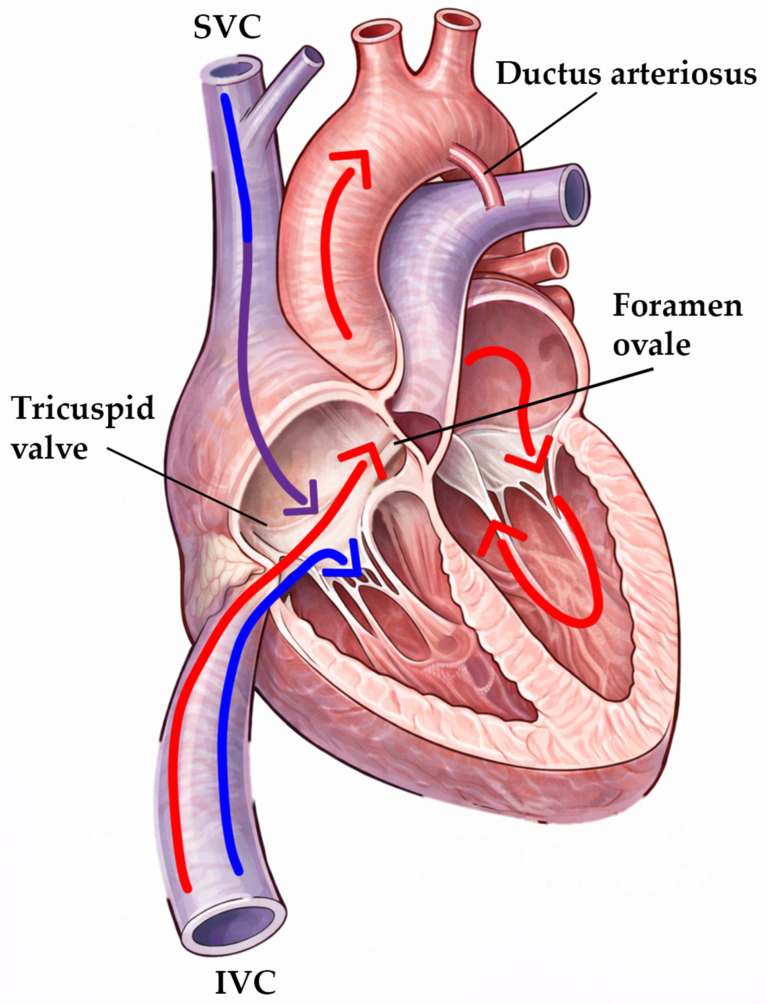
Preferential caval flow streaming in the foetal circulation. Schematic illustrating preferential streaming of inferior vena cava (IVC) blood across the foramen ovale into the left atrium (LA), whereas superior vena cava (SVC) blood predominantly enters the right ventricle via the tricuspid valve, with only a small fraction reaching the LA. This physiological separation supports preferential delivery of relatively oxygenated venous return to the developing brain and myocardium. Stream colour: red—oxygenated blood; blue—deoxygenated blood; violet/purple—mixed blood.

POS is characterised by dyspnoea with objective arterial oxygen desaturation that occurs on assuming the upright position (sitting/standing) and improves with recumbency [10,11,12,13]. In most POS presentations, the dominant abnormality is an intracardiac R–L shunt across a PFO (or less commonly an atrial septal defect (ASD)), often in the absence of sustained pulmonary hypertension or markedly elevated right-sided filling pressures [12,13]. A widely cited mechanistic framework emphasises flow redirection rather than pressure-driven shunting: acquired anatomic distortion (e.g., post-pneumonectomy mediastinal shift, hemidiaphragm elevation/paralysis, aortic elongation/dilatation, and atrial septum stretching) can alter the spatial relationship between the interatrial septum and caval inflow, promoting preferential “streaming” of inferior vena cava (IVC) blood across the PFO, particularly in the upright posture [12,13,14,15]. This concept is biologically plausible in light of foetal flow-streaming physiology (as shown in Figure 1). Lamb experiments using starch granules and contrast injected separately into the superior vena cava (SVC) and IVC demonstrated that SVC-derived flow predominantly traverses the tricuspid valve, with only a small fraction entering the left atrium (LA), whereas IVC-derived flow preferentially streams through the foramen ovale into the left atrium, an arrangement thought to prioritise oxygen delivery to the developing brain and myocardium [16]. This physiology has practical diagnostic implications: in suspected POS (or when streaming is suspected), “site-specific” bubble testing can be diagnostically decisive—upper-limb injection may be weak/negative, whereas femoral/IVC injection can demonstrate a large shunt due to preferential IVC streaming [14,17]. In parallel, identifying shunt physiology (e.g., an elevated alveolar–arterial gradient in otherwise unexplained hypoxaemia) may help prioritise intracardiac shunting early in the diagnostic pathway. Furthermore, POS may be under-recognised when pulse oximetry is not routinely measured in both supine and upright postures [17]. Larger POS series also highlight frequent complex septal morphology (e.g., atrial septal aneurysm, increased septal angulation, and shorter primum–secundum overlap), which may necessitate individualised device selection; a substantial minority may require a non-PFO occluder to achieve effective closure [12]. Across observational cohorts, percutaneous closure is associated with rapid and clinically meaningful improvement in orthostatic oxygenation and symptoms, although persistent/residual shunting is a major correlate of suboptimal response [12,18,19]. Another explanation for absent symptomatic benefit is advanced underlying lung disease complicated by severe pulmonary hypertension (mean pulmonary artery pressure > 50 mmHg) [20].

EID—also described as provoked exercise desaturation (PED)—represents a distinct PFO-associated phenotype in which oxygen desaturation is induced by exertion and typically resolves promptly with rest, often without the posture dependence required for POS. Reports emphasise that EID can be missed by routine resting assessments; deliberate reproduction using ambulatory oximetry, standardised stair/walk protocols, or formal exercise testing is often required, followed by echocardiographic confirmation of an intracardiac shunt. Mechanistically, EID is commonly interpreted as a transient augmentation of R–L shunting during exertion (e.g., increased venous return and dynamic intrathoracic/interatrial pressure relationships), producing inducible rather than persistent hypoxaemia. Interventional data are largely observational, yet multiple reports show improvement or resolution of exertional desaturation and functional limitation after PFO closure; repeat exercise testing often confirms abolition of desaturation, although full physiologic normalisation may not occur in all patients [2,3,9]. A comparison of POS and EID in patients with PFO is provided in Table 1.

## 3. PFO-Mediated Hypoxaemia

In patients with a PFO and no underlying pulmonary pathology, LA pressure typically exceeds RA pressure by 5–7 mmHg [21,22]. When hypoxaemia occurs, it usually reflects intermittent, trigger-dependent R–L interatrial shunting with venous admixture, allowing deoxygenated blood to bypass pulmonary gas exchange and causing arterial desaturation that can appear disproportionate to pulmonary findings. In patients with chronic hypoxemic lung disease, a co-existing PFO-related R–L shunt can further lower arterial oxygenation out of proportion to the primary pulmonary pathology, and separating the relative contributions of parenchymal disease versus intracardiac shunting may be challenging. Nonetheless, PFO-mediated hypoxaemia remains under-recognised and undertreated.

### 3.1. Obstructive Sleep Apnoea (OSA)

Obstructive sleep apnoea (OSA) is common (occurring in approximately 10–15% of adult females and 20–30% of adult males) [23] and produces intermittent hypoxaemia from recurrent upper-airway obstruction with episodes of apnoea/hypopnoea [24]. In patients with a co-existing PFO, these episodes can intermittently augment R–L interatrial shunting, adding venous admixture and worsening systemic desaturation in a trigger-dependent manner, conceptually aligning with other stressors (including exertion) that transiently increase right-sided pressures. Mechanistically, apnoea-related hypoxaemia and hypercapnia increase pulmonary arteriolar resistance with transient rises in pulmonary artery and RA pressures, while large intrathoracic pressure swings during obstructed inspiratory efforts can deform the interatrial septum and facilitate shunting; R–L shunting has been observed during apnoeic episodes but not necessarily while awake [25,26,27,28]. Observational data also suggest a higher prevalence of PFO in OSA and greater provoked desaturation among OSA patients with PFO despite similar resting saturations; in one transoesophageal echocardiography (TOE) study, PFO was detected in 69% of OSA patients versus 17% of controls, and Valsalva provocation produced more pronounced desaturation in those with PFO [25], with other cohorts linking R–L shunt to greater hypoxaemia relative to OSA severity (higher oxygen desaturation index (ODI)/apnoea–hypopnoea index (AHI)) and larger shunt burdens [27,28]. Consistent with haemodynamic dependence, continuous positive airway pressure (CPAP) has been reported to suppress R–L shunting, supporting reversibility when obstructive events and their pressure effects are mitigated [29,30].

### 3.2. Chronic Obstructive Pulmonary Disease (COPD)

Observational data suggest that PFO is common in chronic obstructive pulmonary disease (COPD; reported prevalence: ~45–54%), but not all studies confirm higher rates versus controls; variability likely reflects differences in diagnostic modality (transthoracic echocardiography (TTE) vs. TOE vs. transcranial Doppler (TCD) ultrasonography) and shunt definitions, and TCD cannot distinguish intracardiac from transpulmonary R–L shunts [31,32,33,34].

In severe COPD, a TOE bubble study with Valsalva showed an approximately twofold higher PFO prevalence compared with controls; about half of affected patients had transient oxygen desaturation, correlating with COPD-associated pulmonary hypertension [32]. A TTE bubble-study cohort similarly reported lower arterial oxygen saturation, a shorter 6 min walk distance, and more severe disease in COPD patients with PFO versus those without [35]. In contrast, a small study with COPD patients in stage Global Initiative for Chronic Obstructive Lung Disease (GOLD) II using TCD found no difference in exercise capacity or 6 min walk performance, although interpretation is limited by sample size, lack of controls, and few large shunts [36].

Importantly, invasive haemodynamic data in COPD underscore that exertional hypoxaemia and pulmonary vascular responses are not tightly coupled, complicating attribution of desaturation to an intracardiac shunt alone. In a right-heart catheterisation study of 17 COPD patients with no or only mild hypoxaemia at rest (mean resting partial pressure of arterial oxygen (PaO_2_): 10.6 ± 1.1 kPa), pulmonary artery pressure (PAP) rose substantially even during low-intensity exercise intended to approximate activities of daily living (mean pulmonary arterial pressure: 35.0 ± 2.2 mmHg), meeting criteria for exercise-induced pulmonary hypertension in 11/17 (65%) despite only a modest mean fall in PaO_2_ (to 9.7 ± 0.4 kPa). Although exercise PAP and PaO_2_ were inversely correlated, discordant patterns were frequent: exercise-induced pulmonary hypertension occurred in some patients without hypoxaemia and, conversely, was absent in some with exercise-induced hypoxaemia; similarly, an elevated pulmonary vascular resistance index without exercise hypoxaemia was observed in 35% [37].

These observations support a multifactorial model in which pulmonary vascular remodelling and ventilatory–perfusion mismatch may contribute to exertional desaturation independently of (and potentially in addition to) PFO-mediated R–L shunting, reinforcing the need for careful physiological phenotyping when considering PFO closure in COPD.

### 3.3. High-Altitude Pulmonary Oedema (HAPO)

High-altitude pulmonary oedema (HAPO) is a non-cardiogenic syndrome triggered by rapid ascent (typically above 2500 m). Its hallmark is an exaggerated rise in PAP that precedes oedema formation, with reported mean pressures in the range of 35–55 mmHg. Characteristic early findings include elevated pulmonary capillary pressure and oedema fluid that is protein- and red blood cell-rich, without evidence of inflammation; impaired alveolar fluid clearance may further contribute to oedema development [38]. In the presence of a PFO, low oxygen tension at altitude may further augment PAP via reactive vasoconstriction and initiate or amplify R–L shunting. The resulting venous admixture worsens systemic desaturation, reduces mixed venous oxygen content, and may further increase PAP, creating a self-perpetuating spiral of hypoxaemia and pulmonary oedema [39,40].

Observational data support a clinically relevant association. In a TOE study, climbers who developed HAPO had a markedly higher prevalence of PFO than resistant controls, both at low altitude (56% vs. 11% at 550 m; *p* = 0.004) and at high altitude (69% vs. 16% at 4559 m; *p* = 0.001). Among those with HAPO, a large PFO was associated with more severe arterial hypoxaemia (mean oxygen saturation: 73% vs. 83%; *p* = 0.001) [41]. R–L shunting through a PFO has also been linked to blunted ventilatory acclimatisation at altitude, which may further increase HAPO susceptibility [42].

### 3.4. Ebstein Anomaly

Ebstein anomaly is characterised by deformities of the anterior tricuspid leaflet with atrialisation of the right ventricle, and apical displacement of the functional tricuspid annulus towards the right ventricular apex [43,44,45]. Because the greatest displacement is typically septal, the tricuspid regurgitant jet is preferentially directed towards the interatrial septum and may stream across an interatrial communication (PFO or ASD), which is common in this condition, producing R–L shunting and systemic desaturation [43,45].

As tricuspid regurgitation can worsen with exertion, Ebstein anomaly represents a structural substrate in which exertional hypoxaemia may become apparent or be accentuated by increased shunt streaming across a PFO/ASD [43,46]. In appropriately selected patients, after careful haemodynamic assessment, closure of a PFO/ASD may relieve hypoxaemia; where severe tricuspid regurgitation is present, tricuspid valve surgery is generally undertaken with concomitant PFO/ASD closure [45,46].

## 4. Exercise-Induced Arterial Hypoxaemia in Otherwise Healthy Individuals

Exercise-induced arterial hypoxaemia (EIAH) refers to a reproducible fall in arterial oxygenation during heavy exercise in otherwise healthy people, most often described in endurance-trained athletes. In the classic treadmill study of highly trained runners by Dempsey and colleagues, responses were heterogeneous: some athletes maintained arterial oxygen tension close to resting values, whereas others developed substantial decrements—to <75 mmHg in several cases and <60 mmHg in a minority—typically emerging after the initial phase of heavy constant-load exercise [47]. Non-invasive definitions commonly use pulse oximetry, for example, a ≥4% sustained fall in peripheral oxygen saturation (SpO_2_) from rest during the latter stages of incremental exercise; severity has been pragmatically stratified by SaO_2_ as mild (95–93%), moderate (93–88%), and severe (<88%) [48].

Pathophysiologically, EIAH reflects pulmonary gas exchange limitation under extreme metabolic and haemodynamic stress, leading to an increased alveolar–arterial oxygen gradient, with several mechanisms contributing in variable proportions between individuals [49]. First, exceptionally high cardiac outputs shorten pulmonary capillary red blood cell transit time; when transit becomes very brief—particularly as mixed venous oxygen tension falls during heavy work—complete oxygen equilibration may not occur (diffusion disequilibrium) [47,49]. Second, heavy exercise may accentuate ventilation–perfusion maldistribution, widening the alveolar–arterial gradient and lowering SaO_2_. Third, some athletes demonstrate a relatively constrained ventilatory response for a given metabolic load (whether due to mechanical ventilatory constraints, respiratory muscle work, or inter-individual variability in ventilatory drive), reducing alveolar oxygen tension and predisposing to desaturation [49]. While transient intrapulmonary shunt fractions may contribute in some individuals, current syntheses generally attribute EIAH in healthy subjects primarily to a combination of ventilation–perfusion inequality, diffusion limitation during high-flow states, and ventilatory constraints, with the relative contribution of each mechanism varying between individuals [48,49].

Clinically, EIAH is important in the differential diagnosis of exertional desaturation because it represents a physiological background phenomenon in healthy—often endurance-trained—individuals at very high exercise intensities. Accordingly, when an athlete desaturates during exertion, a PFO may be an incidental finding rather than the primary driver; attribution to PFO-mediated EID should, therefore, be made only after objective demonstration of inducible intracardiac R–L shunting temporally linked to the desaturation episode.

## 5. Prevalence

In the absence of broader prospective datasets, the only published prospective prevalence estimate for EID in referred PFO patients comes from a single-centre cohort published in 2012.

In this study, Devendra et al. enrolled 50 consecutive adults with newly diagnosed PFO referred for cardiovascular assessment and applied a standardised pulse oximetry protocol across postural manoeuvres and stair-climbing exertion. EID was defined as a sustained reduction in oxygen saturation of at least 8% from baseline to a value ≤ 90% [3]. For interpretability, we use this protocolised threshold when discussing prevalence estimates; however, in clinical practice and case-based reports, exertional desaturation may remain clinically relevant when it is reproducible and temporally linked to provoked intracardiac R–L shunting, even if the nadir does not reach ≤90%.

EID was identified in 17/50 patients (34%). Baseline characteristics were broadly comparable between those with and without EID; the overall cohort had a mean age of 46 ± 17 years, was predominantly female (74%), and included substantial proportions with migraine (30%) and prior transient ischaemic attack (TIA)/stroke (48%). Notably, EID was often unrecognised prior to protocolised testing. Only 6/50 patients were referred primarily for oxygen desaturation, yet 4/6 met criteria for EID, and 13/17 EID cases were detected in patients without a documented history of desaturation.

Overall, 21/50 patients (42%) underwent PFO closure (13 with EID; eight without EID), predominantly percutaneous (19/21) with a minority being surgical (2/21). Among percutaneous procedures, several occluder systems were used: Helex (16/19), STARFlex (1/19), CardioSeal (1/19), and an Amplatzer cribriform device (1/19).

Among EID patients with available 3-month reassessment after closure (10/13), the mean exercise-related saturation drop decreased from 12.6 ± 3.0% pre-closure to 2.5 ± 1.6% post-closure (mean improvement: 10.1 ± 4.2%; *p* < 0.001), accompanied by NYHA class improvement from a median of 3 (IQR 2–3) to 1 (IQR 1–2) (median improvement: 1.5 classes; *p* = 0.008). Minor residual shunting was documented in five patients post-closure, but in this cohort, it was not reported to negate improvement in EID.

While the observed prevalence is likely influenced by referral selection and should not be extrapolated to unselected PFO populations, this study provides the best available prospective benchmark and supports incorporating simple exertional oximetry (e.g., stair climbing) when evaluating suspected exertional hypoxaemia in the setting of PFO [3].

## 6. Pathophysiological Mechanisms

EID in the setting of a PFO is best understood as an intermittent, provocation-dependent R–L shunt that emerges when exertion recruits haemodynamic and flow-dynamic conditions sufficient to open the PFO and redirect systemic venous blood into the LA [3]. Conceptually, this can be viewed as an exertional analogue of the “anatomy plus functional trigger” paradigm outlined for POS (Section 2), but with the trigger dominated by exercise-related physiology rather than posture alone. This aligns with the framework used for cardiac POS syndrome, in which clinically relevant R–L shunting typically requires both an anatomical interatrial communication (PFO/ASD) and a superimposed functional factor that either redirects venous flow towards the defect or intermittently reverses the usual left-to-right interatrial pressure gradient [50]. Under resting conditions, LA pressure typically exceeds RA pressure, which should limit sustained R–L shunt; EID, therefore, implies a transient reversal or functional “overriding” of this gradient during exercise, most plausibly through abrupt increases in venous return (including the skeletal muscle pump during stair climbing), exaggerated intrathoracic pressure swings, and short-lived changes in atrial filling and compliance that favour momentary R–L passage [3,16]. Importantly, several datasets indicate that resting assessment can substantially underestimate clinically relevant shunting. In a large hypoxaemia-referred cohort undergoing exercise saline-contrast echocardiography, moderate-or-greater R–L shunt was common during exertion (48/59), whereas only a minority demonstrated comparable shunt severity at rest (11/48), supporting a mechanism in which exercise recruits or amplifies shunt physiology rather than merely revealing a fixed defect [8].

**Figure 2:**
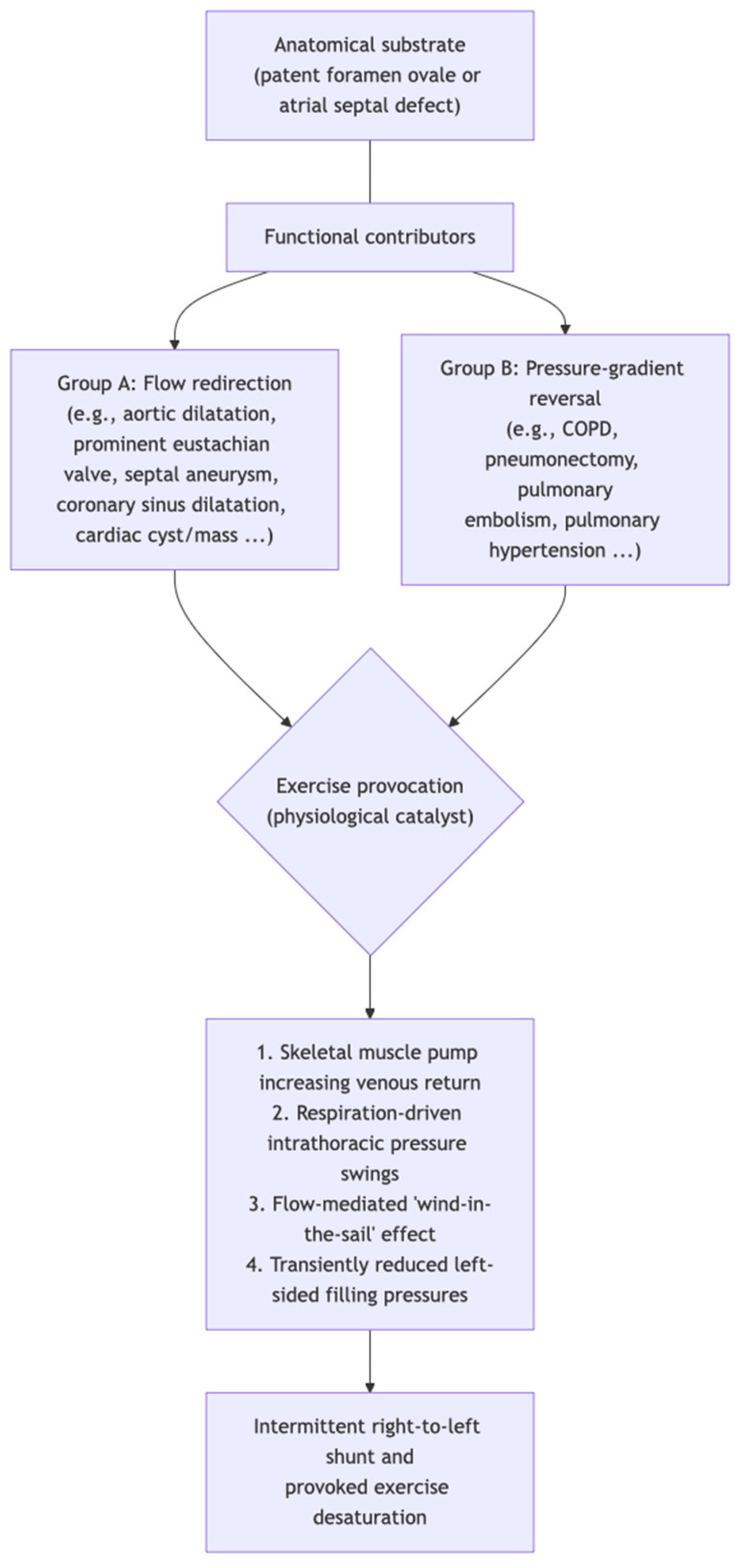
Pathophysiological framework for provoked exercise desaturation.

Beyond pressure gradients, contemporary models emphasise “non-pressure-driven” components, whereby altered RA flow streaming and vortex formation direct IVC blood towards the fossa ovalis and mechanically facilitate separation of the septum primum (a flow-mediated “wind-in-the-sail” effect), enabling R–L shunt even without sustained elevation of RA pressure [16]. Knapper et al. usefully group functional contributors into (i) “flow redirection” mechanisms—where anatomical distortion or structures preferentially channel venous return (often IVC-derived) towards the interatrial communication—and (ii) “pressure-gradient reversal” mechanisms, in which conditions that increase right-sided pressures or pulmonary vascular resistance facilitate episodic R–L transit [50]. Anatomical and age-related modifiers likely determine individual susceptibility by altering the spatial relationship between the venae cavae, atrial septum and aorta; proposed contributors include interatrial septal remodelling and aortic dilatation/elongation that progressively redirect venous inflow towards the PFO, rendering patients asymptomatic for years before EID manifests later in life [2,16]. Consistent with this, symptoms may present late and be precipitated by an inciting cardiac or extracardiac event associated with mechanical distortion—such as pneumonectomy with mediastinal shift, aortic root aneurysm or elongation, kyphosis, or unilateral diaphragmatic paralysis—which can alter the position of the interatrial septum relative to the inferior vena cava and thereby substantially increase shunt propensity [51]. Exercise may further promote R–L shunt by lowering systemic vascular resistance and left-sided filling pressures in some individuals, thereby reducing the “closing” force on the interatrial flap while venous return rises [9]. Physiological observations from CPET are consistent with an acute shunt phenomenon, including exertional desaturation accompanied by abrupt ventilatory changes and alterations in end-tidal gases [2]. Collectively, these mechanisms support a unifying concept: EID represents a dynamic interaction between exertion-related haemodynamics, respiration-driven intrathoracic pressure variation, flow-directed streaming across a permissive PFO anatomy, and age-/geometry-related changes that favour R–L transit, often in the absence of resting pulmonary hypertension or a large resting shunt [2,3,8,16]. The pathophysiological framework is illustrated in the algorithm presented in Figure 2.

## 7. Diagnostic Approach

EID related to PFO is frequently overlooked because resting observations may be normal; diagnostic evaluation should, therefore, prioritise reproducing desaturation under supervision and subsequently demonstrating an inducible R–L shunt. A pragmatic first step is simple exertional oximetry (corridor walking or stair climbing) performed in a clinic. However, nomenclature and definitions are not fully standardised: in the only prospective, protocolised series, oxygen saturation was recorded during postural manoeuvres and a standardised stair protocol (ascent and descent of four flights), with “EID” or “PED” defined as above [3]. This aligns with contemporary bedside practice emphasising “in situ” observation of desaturation during walking when symptoms occur.

Once exertional hypoxaemia is confirmed, alternative cardiopulmonary causes should be actively excluded (e.g., pulmonary embolism, parenchymal lung disease, ventilatory limitation, etc.), recognising that many patients have mixed-aetiology dyspnoea. In the largest hypoxaemia-focused closure cohort, patients were evaluated for resting or exertional hypoxaemia (6 min walk test or exercise contrast echocardiography), and those with suspected pulmonary contributors underwent structured pulmonary investigation [8]. At this stage, it is also important to consider “mimics” of intracardiac shunt syndromes; patients may develop POS-like physiology (and clinically similar desaturation patterns) without a cardiac aetiology. In such cases, the anatomical substrate is typically a pulmonary arteriovenous malformation or severe ventilation–perfusion mismatch, with superimposed functional factors that increase flow through malformations or worsen mismatch; chronic liver disease is a notable association via hepatopulmonary syndrome, in which worsening liver disease can parallel worsening orthostatic desaturation. Although framed for POS, the same principle applies to suspected PFO-related EID; exertional desaturation should not be attributed to a PFO until relevant pulmonary shunt/ventilation–perfusion mismatch processes have been reasonably excluded [50].

Confirmation of the mechanism requires contrast testing targeted to the provoked R–L shunt. TTE with agitated saline (ideally with an effective end-expiratory Valsalva) is a practical first test; TOE provides superior anatomical definition but may still be falsely negative if sedation limits Valsalva performance, and TCD is sensitive but cannot localise the shunt [52]. Because exertion is a key trigger, negative resting studies—including a “negative” Valsalva—should not be considered definitive. Mechanistically, Devendra et al. emphasise that LA pressure usually exceeds RA pressure, such that R–L shunting requires a provoking circumstance; rigorous exertion may generate a larger and more sustained venous return/RA loading than a brief or suboptimal Valsalva, transiently overcoming the usual interatrial gradient [3]. Moreover, exercise can recruit flow-dynamic drivers beyond pressure, including altered RA streaming/vortices that direct IVC blood towards the fossa ovalis and facilitate PFO opening (“wind-in-the-sail”), with respiro-phasic effects amplified during exertion [16]. In line with this, exercise protocols are particularly informative: Fenster et al. used Valsalva, cough, or exercise when resting injections were negative, and demonstrated that exertion commonly unmasks clinically relevant shunting that is not evident at rest [8].

**Figure 3:**
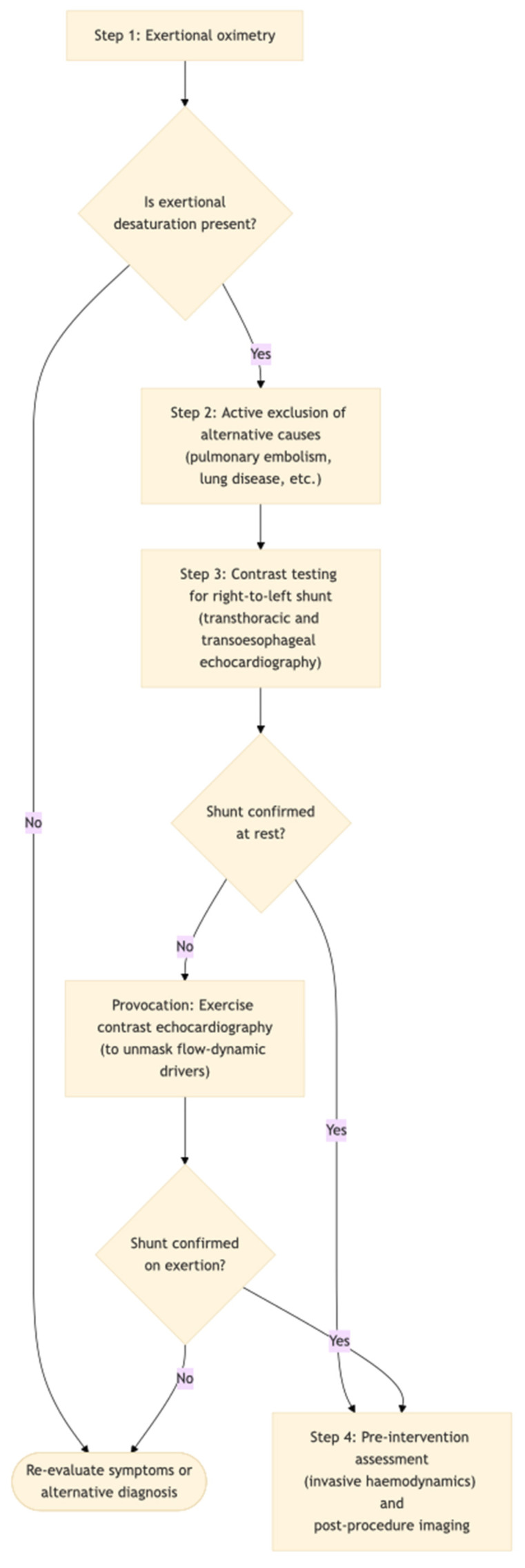
Proposed diagnostic algorithm for the evaluation of provoked exercise desaturation in patients with PFO.

Where closure is contemplated, invasive haemodynamic assessment is important to exclude significant pulmonary hypertension and to reduce the risk of misattributing multifactorial hypoxaemia to R–L shunting alone [8]. Post-procedure, repeat contrast imaging is essential to document residual shunt, given evidence in hypoxaemia syndromes that significant residual shunting is a major determinant of non-response [19]. The proposed diagnostic algorithm for the evaluation of PED in patients with PFO is presented in Figure 3.

## 8. Does the Closure of the PFO Help in Hypoxaemia Syndromes?

Across hypoxaemia syndromes attributed to PFO-mediated R–L shunting—including POS and exertional desaturation—the available evidence (predominantly observational) supports a clinically meaningful benefit from closure in appropriately selected patients. Importantly, the indication appears uncommon in routine practice. In a Belgian multicentre experience spanning three hospitals (January 2000–September 2018), only 16 patients were identified in whom dyspnoea and/or hypoxaemia was the primary indication for percutaneous PFO closure, despite 1287 total closures performed over the same period [51].

The most informative dataset remains the single-centre retrospective series by Fenster et al., who analysed 97 patients (2004–2009) referred for systemic hypoxaemia and dyspnoea deemed disproportionate to underlying lung disease, all with R–L shunt on contrast echocardiography. Despite frequent pulmonary comorbidity (67%) and oxygen dependence (42%), patients were selected to avoid advanced pulmonary vascular disease; closure required symptomatic hypoxaemia, demonstrable intracardiac R–L shunt at intracardiac echocardiography, prior evidence of desaturation at rest or with exertion, and absence of significant pulmonary hypertension (mean pulmonary artery pressure > 45 mmHg and pulmonary vascular resistance > 5 Wood units). Percutaneous closure was mechanically effective (procedural success: 96/97 (99%)) and was associated with clinical success in 68/97 (70%), driven mainly by improvement in NYHA class (49/97 (51%); *p* < 0.001), with a further 17/97 (18%) reporting symptomatic improvement without NYHA class change. Among patients on chronic supplemental oxygen (41/97), oxygen requirements decreased in 14/41 (34%; *p* < 0.001), including complete discontinuation in four patients. Importantly, this cohort illustrates why benefit may be missed if assessment is limited to resting studies: 22/97 (23%) had no R–L shunt at rest but became positive with Valsalva, cough, or exercise, and among those undergoing exercise saline contrast echocardiography (59/97), moderate-or-greater shunt was present in 48/59 during exercise, whereas only 11/48 showed moderate-or-greater shunt at rest. In a small pre/post subgroup (*n* = 12), EID decreased in 7/12 (mean: 9 ± 3% to 7 ± 5%), and treadmill exercise time improved in 8/12, supporting a physiological signal beyond subjective endpoints. Nonetheless, Fenster et al. explicitly note that closure may yield incremental improvements without complete normalisation—particularly in medically complex patients—underscoring the attribution challenge when pulmonary disease coexists [8].

Complementing these data, the multicentre series by De Cuyper et al. specifically focused on patients closed for dyspnoea and/or reduced oxygen saturation at rest and during exercise: closure was successful in 15/16, and all patients reported immediate subjective improvement in dyspnoea at device deployment, accompanied by improved upright oxygenation on room air (from 90 ± 6% pre-closure to 94 [92–97]% post-closure; *p* < 0.05) with sustained benefit over a median follow-up of 36 [12,13,14,15,16,17,18,19,20,21,22,23,24,25,26,27,28,29,30,31,32,33,34,35,36,37,38,39,40,41,42,43] months (range: 0–14 years); post-procedural questionnaires also indicated improvement in dyspnoea and quality of life, and reported deaths were unrelated to the procedure [51].

Complementary evidence in the more specific phenotype of EID comes from the prospective cohort by Devendra et al., in which PFO closure in PED improved the exercise-related saturation drop by 10.1 ± 4.2% at 3 months (*p* = 0.001) and improved NYHA functional class by a median of 1.5 classes (*p* = 0.008); minor residual shunting did not appear to abrogate benefit in those with follow-up [3].

The case-based literature also supports rapid functional recovery after closure when exertional desaturation is clearly linked to R–L shunting, including a report of a 79-year-old with walking-related desaturation to 87% and a large PFO with bidirectional shunt who had resolution of desaturation and improved functional capacity shortly after device closure [2]. At the same time, individual cases illustrate that symptomatic benefit may exceed measurable oxygenation normalisation. In a post-COVID presentation with exertional desaturation to 81% in the 6 min walk test, closure eliminated residual shunt and markedly improved quality of life, yet follow-up exertional saturation remained reduced (88%), consistent with multifactorial hypoxaemia in some patients [9].

Finally, contemporary registry data in POS/severe hypoxaemia reinforce both the effectiveness and the importance of residual shunt: in a monocentric Strasbourg registry (2010–2024), 50/264 closures were performed for POS or severe hypoxaemia, with improvement in oxygenation and dyspnoea in 36/50 (72%); significant residual shunt (12% at follow-up) was the main predictor of failure and was associated with device disc separation > 5.05 mm [19].

Importantly, across published series and in our illustrative case, improvement in exertional oxygenation should not be assumed to translate into a measurable increase in maximal aerobic capacity; closure may predominantly improve gas exchange and symptom burden, while peak work and maximal oxygen uptake can remain unchanged in some patients.

Regarding practical selection considerations (derived from the reviewed evidence), PFO closure for hypoxaemia syndromes should generally be considered only when

- Reproducible exertional and/or postural arterial desaturation is documented by supervised oximetry, CPET, or a standardised walk/stair protocol;
- An intracardiac R–L shunt is objectively demonstrated and is inducible or clearly accentuated by provocation (Valsalva, cough, posture, or exercise) on contrast echocardiography (±TCD where appropriate);
- Dominant alternative explanations for hypoxaemia have been reasonably excluded (e.g., parenchymal lung disease, pulmonary embolism, pulmonary arteriovenous malformations, hepatopulmonary syndrome, or severe ventilation–perfusion mismatch);
- Haemodynamic assessment excludes significant pulmonary hypertension that would render closure unsafe or unlikely to help.

Where benefit is expected, it may manifest primarily as improved oxygenation and symptom burden rather than a consistent increase in maximal exercise capacity.

Taken together, these data support PFO closure as a potentially effective intervention for selected hypoxaemia syndromes mediated by R–L shunting, while emphasising the need for provoked/exercise assessment to document clinically relevant shunting and for meticulous anatomical/technical optimisation to minimise residual shunt [3,8,19].

## 9. Case Study

### 9.1. History and Physical Examination

We report a 50-year-old man with arterial hypertension who sustained an ischaemic stroke in 2020, after which a PFO was diagnosed. He was a never-smoker with no known underlying lung disease. He was enrolled in the MEASURE-PFO study, the aim of which was to quantify R–L shunt in patients with PFO using an original thermodilution method and to assess its relationship with interatrial septal morphology and cardiopulmonary performance before and after PFO closure. As part of the study protocol, he underwent CPET before and after percutaneous closure; clinically relevant arterial desaturation was documented during the pre-closure CPET.

### 9.2. Imaging

TOE demonstrated an aneurysmal interatrial septum with marked excursions and a clearly defined PFO tunnel. Agitated-saline contrast imaging during a Valsalva manoeuvre showed a PFO tunnel height of 4–5 mm and a large R–L passage of microbubbles from the RA to the LA, consistent with a substantial provocable shunt.

### 9.3. Indication for Intervention and Physiological Assessment

**Figure 4:**
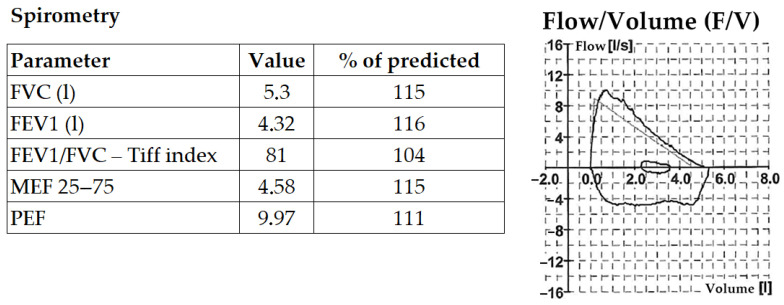
Baseline spirometry demonstrating normal ventilatory function. Pre-procedural spirometry was within normal limits, with no evidence of obstructive or restrictive ventilatory impairment. FVC, forced vital capacity; FEV1, forced expiratory volume in 1 s; FEV1/FVC (Tiffeneau index), ratio of forced expiratory volume in 1 s to forced vital capacity; MEF 25–75, mean expiratory flow between 25% and 75% of FVC (reflecting small airway function); PEF, peak expiratory flow.

**Figure 5:**
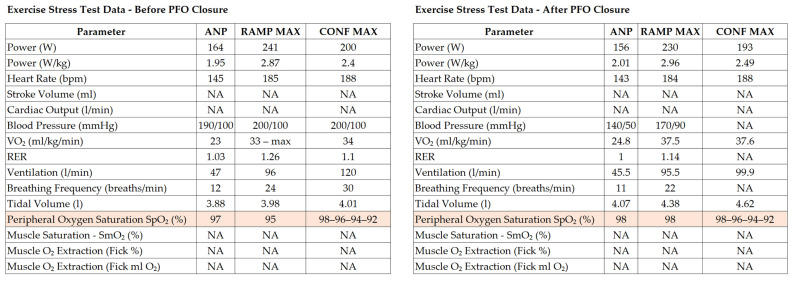
Cardiopulmonary exercise testing before and after PFO closure. Before PFO closure, exercise provoked a progressive fall in oxygen saturation (98% → 92%); after closure, oxygen saturation remained stable (98% → 97%), with comparable peak workload and heart rate. ANP, anaerobic threshold; RAMP MAX, maximum value achieved during the incremental ramp protocol; CONF MAX, maximum value achieved during the confirmatory phase performed after recovery; VO_2_, oxygen uptake; RER, respiratory exchange ratio; NA, not available. The complete cardiopulmonary exercise testing dataset (including Wasserman plots, ventilatory and gas-exchange indices, and calorimetry outputs) is available from the authors upon reasonable request.

PFO closure was indicated for secondary prevention following paradoxical embolism. Pre-procedural CPET showed normal spirometry and preserved aerobic capacity (with a maximal oxygen uptake (VO_2_max) of 33 mL/kg/min, within the expected range for an untrained male). During incremental exercise, oxygen saturation progressively declined on forehead reflectance oximetry (98% → 96% → 94% → 92%). Oxygen saturation was measured using a head reflectance sensor to minimise artefact from finger perfusion limitation during high-intensity exercise. The protocol comprised a ramp phase (target duration: 8–12 min) followed—after recovery—by a confirmatory stage intended to impose higher haemodynamic stress and maximise the likelihood of reproducing exertion-provoked desaturation. No additional reproducible abnormalities in ventilatory indices or end-tidal gas measures were documented in this case; the salient physiological finding was exertion-provoked arterial desaturation on a background of preserved spirometry and aerobic capacity. Baseline spirometry demonstrating normal ventilatory function is shown in Figure 4. Nevertheless, clinicians should be aware that CPET can sometimes suggest an inducible R–L shunt through a characteristic pattern: a sudden and sustained fall in end-tidal carbon dioxide pressure (PETCO_2_) with a concomitant rise in ventilatory equivalent for carbon dioxide (VE/VCO_2_) and ventilatory equivalent for oxygen (VE/VO_2_, reflecting increased minute ventilation), often accompanied by an increase in end-tidal oxygen pressure (PETO_2_) and a fall in SpO_2_. When such changes occur, targeted investigation for inducible R–L shunting (e.g., contrast echocardiography with provocation, and—where uncertainty persists—arterial blood gases or invasive CPET to assess oxygenation and the alveolar–arterial oxygen gradient) may be informative [53]. CPET findings before and after PFO closure are presented in Figure 5.

### 9.4. Intervention

The patient underwent uncomplicated percutaneous PFO closure using a 25/25 mm Ceraflex device and was discharged the following day. During the same procedure, R–L shunt magnitude was assessed using an investigational thermodilution-based approach (Inntherm^®^; Hradec Králové, Czech Republic, research-use only, not commercially available). Standard haemodynamic catheterisation required both femoral arterial (1x 4 French access) and femoral venous access (1x 4 French and 1x 6 French access), and intracardiac, aortic, and pulmonary artery pressures were recorded using a dedicated haemodynamic recording system. In addition to shunt assessment, systemic blood flow (SBF) and pulmonary blood flow (PBF) were quantified, enabling calculation of pulmonary-to-systemic flow ratio (Qp/Qs) and shunt fractions.

#### Catheter Positions and Measurement Phases (as Shown in Figure 6, Figure 7 and Figure 8)

(i) SBF (systemic thermodilution): a thermosensing probe was advanced retrogradely via the femoral artery into the descending thoracic aorta (just distal to the aortic arch) to record the aortic temperature–time curve, while a pigtail catheter was positioned in the left ventricle for cold-saline indicator injection (multiple technically acceptable curves were recorded and averaged).
(ii) PBF (pulmonary thermodilution): a guiding catheter was positioned in the pulmonary artery, and the thermosensing probe was deployed into the pulmonary artery to record the pulmonary temperature–time curve; the indicator (cold saline) was injected via a pigtail catheter positioned in the inferior vena cava near its junction with the RA (again, replicate curves were acquired and averaged).
(iii) R–L shunt (rest and provocation): the thermosensing probe was returned to the descending thoracic aorta (same aortic position as in phase (i)), and 10 mL of chilled saline was injected into the inferior vena cava near the RA. In this configuration, an early aortic temperature deflection indicates direct R–L transit (intracardiac shunt), whereas the later component reflects transpulmonary passage (as illustrated in Figure 7). Measurements were obtained at rest and during Valsalva provocation, with Valsalva timing guided by the invasive arterial pressure tracing to ensure a consistent haemodynamic trigger (Figure 7 and Figure 8).

Using this configuration, the provoked R–L shunt flow was 0.75 L/min (750 mL/min) with a Qp/Qs of 0.88 during Valsalva (Figure 8). Following device deployment, repeat CPET showed resolution of EID, without a statistically significant change in exercise tolerance (work performed).

**Figure 6:**
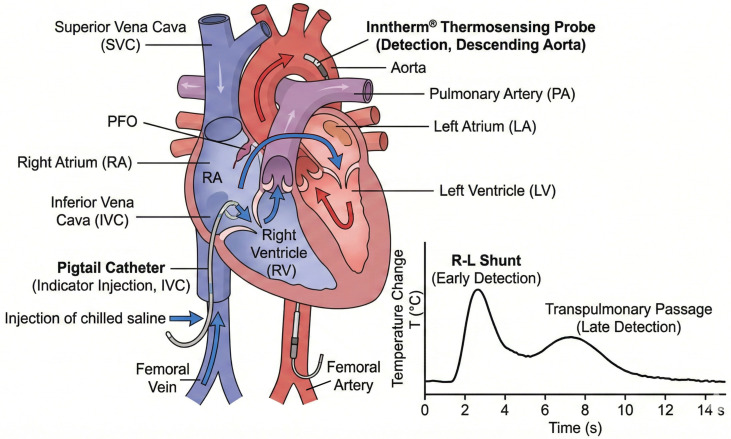
Schematic of the Inntherm^®^ thermodilution set-up for provoked R–L shunt assessment. Schematic showing the catheter configuration for R–L shunt measurement, with chilled-saline indicator injection via a pigtail catheter positioned in the IVC (near the RA junction) and simultaneous temperature–time recording by a thermosensing probe positioned in the descending thoracic aorta just distal to the aortic arch.

**Figure 7:**
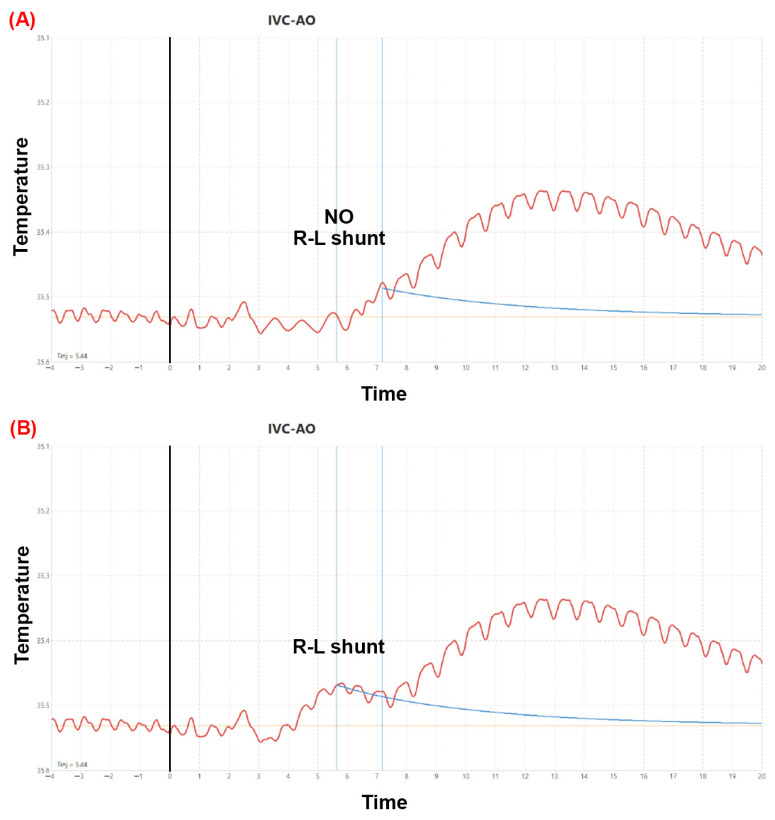
Representative temperature–time (thermodilution) patterns for absence and presence of intracardiac R–L shunt. (**A**) No R–L shunt: single temperature deflection peak reflecting transpulmonary passage only. (**B**) R–L shunt present: biphasic curve with an early temperature deflection indicating direct intracardiac R–L transit and a later component reflecting transpulmonary passage.

**Figure 8:**
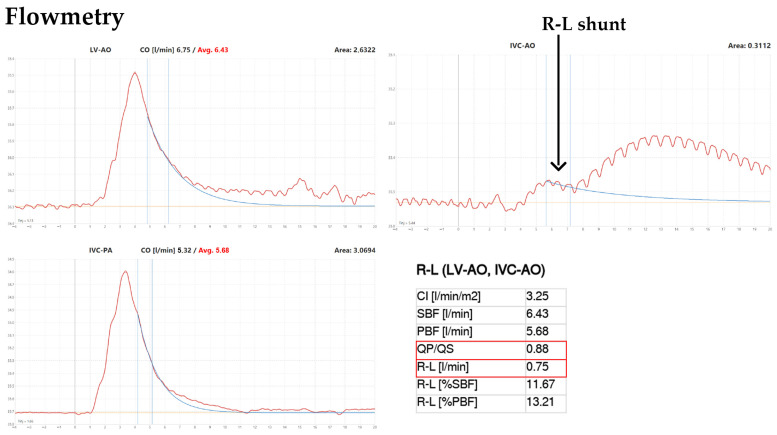
Catheter-based thermodilution assessment of systemic and pulmonary flow with quantification of a provoked R–L shunt across a PFO. Using the Inntherm^®^ thermodilution system during femoral cardiac catheterisation, systemic and pulmonary blood flow were quantified by indicator-dilution measurements, and a provoked R–L shunt was assessed by injecting 10 mL of chilled saline into the inferior vena cava near the RA junction with simultaneous temperature–time recording in the descending thoracic aorta during a standardised Valsalva manoeuvre guided by invasive arterial pressure tracing. From these measurements, pulmonary-to-systemic flow ratio (Qp/Qs) and R–L shunt flow were derived. The figure demonstrates a Valsalva-provoked R–L shunt of 0.75 L/min (750 mL/min) with Qp/Qs of 0.88.

### 9.5. Learning Points

This case integrates the diagnostic and mechanistic principles outlined above and highlights several practical messages. First, EID may be missed by routine resting assessment; in this patient, the key abnormality emerged only during structured exertional testing, reinforcing the value of deliberate provocation (e.g., CPET or supervised walk/stair oximetry) followed by targeted shunt assessment. Second, the concordance of (i) reproducible exertional desaturation on CPET and (ii) a large provokable R–L shunt on contrast TOE supports a causal link between exertion and intracardiac shunting and illustrates why a comprehensive evaluation should include provocation when resting studies are non-diagnostic. Where available, CPET may provide supportive, time-linked clues to inducible R–L shunting; however, the absence of characteristic ventilatory/end-tidal gas patterns does not exclude inducible shunting when exertional desaturation is reproducible (see Section 9.3) [53]. Third, procedural catheter-based quantification of shunt magnitude under provocation provided an additional physiological correlate and may be helpful in selected cases when symptom attribution is challenging or when coexisting cardiopulmonary contributors are suspected. Finally, complete abolition of exertional desaturation after closure—despite no statistically significant change in maximal aerobic capacity or peak workload—underscores that PFO closure may primarily improve gas exchange and symptom burden rather than maximal exercise capacity, and supports closure as a targeted therapy when inducible R–L shunt is demonstrated and alternative causes of exertional hypoxaemia have been reasonably excluded. Consistent with the broader literature, improvement in oxygenation should not be assumed to translate into an increase in VO_2_max, particularly in untrained individuals or those with coexisting cardiopulmonary limitations. In patients with larger shunt burdens, any improvement in peak work may relate to multiple factors and should not be assumed to represent improved oxidative capacity.

## 10. Future Directions

Future work should prioritise prospective, multicentre phenotyping of exertional hypoxaemia in patients with PFO, incorporating standardised exertional oximetry and/or CPET alongside provoked contrast testing to define the prevalence and clinical spectrum of inducible R–L shunting beyond referral-selected cohorts. A key prerequisite is a consensus operational definition of PFO-related EID, including a standardised provocation protocol and reproducibility criteria, together with clinically meaningful oxygen saturation thresholds. Mechanistic studies are needed to disentangle pressure-dependent versus flow redirection mechanisms during exercise, ideally integrating exercise haemodynamics with imaging approaches capable of resolving RA flow streaming. Quantitative shunt metrics also warrant validation. Invasive thermodilution-based estimates, where feasible, should be correlated with contrast shunt grading, continuous oxygen saturation profiles during provocation, symptom burden, and patient-reported outcomes to identify clinically meaningful thresholds. Finally, interventional evidence remains limited; pragmatic registries and comparative studies should evaluate anatomical predictors of response, quantify the impact of residual shunts on exertional oxygenation, and define long-term outcomes using paired pre-/post-closure objective endpoints and validated measures of dyspnoea, quality of life, and oxygen requirement, with CPET parameters included where feasible.

## 11. Conclusions

EID represents an uncommon but clinically important and potentially reversible manifestation of PFO in which exertion recruits haemodynamic and flow-directional conditions that permit inducible R–L shunting. Because resting oxygenation and resting shunt assessment may be normal, diagnosis requires deliberate reproduction of desaturation (simple exertional oximetry or CPET) followed by contrast assessment under provocation, while systematically excluding pulmonary mimics and competing mechanisms. Although the evidence base is predominantly observational, available cohorts and case literature consistently indicate that percutaneous closure can abolish or substantially reduce exertional desaturation and improve functional status in appropriately selected patients; residual shunting appears to be a key determinant of suboptimal response. Finally, clinicians should interpret post-closure physiological endpoints with caution. Normalisation of oxygen saturation during exercise is a robust marker of mechanistic success, but it does not necessarily predict improvement in peak workload or maximal oxygen uptake, particularly in untrained individuals or those with coexisting cardiopulmonary limitations.

## Figures and Tables

**Table 1:** Comparison of POS and EID in patients with PFO.

POS vs. EID	Platypnoea–Orthodeoxia Syndrome	Exercise-Induced Desaturation
Primary trigger	Postural change (supine to upright)	Physical exertion
Dominant mechanism	Flow redirection/streaming (often IVC) due to anatomic distortion; not necessarily pressure-driven	Transient exercise-provoked augmentation of R–L shunting (short-lived changes in RA pressure and/or flow dynamics)
Haemodynamic state	Static (gravity-dependent)	High flow/high pressure (exertional)
Resting arterial oxygen saturation (SaO_2_)	Decreased (in upright position)	Typically normal
Diagnostic key	Positional pulse oximetry	Exercise testing (CPET or stair climbing)
Clinical resolution	Relieved by recumbency	Relieved by rest
Shunt behaviour at rest	May be demonstrable at rest (often accentuated upright); can be present despite normal pressures	Often minimal/absent at rest; inducible during exertion (documented in catheterisation/exercise protocols in case reports)
Practical diagnostic pitfalls	Oximetry often measured only supine; injection site matters (upper limb may miss IVC-streaming shunt)	Resting evaluation may be normal; requires deliberate exercise provocation (e.g., stair/walk oximetry or exercise spirometry/CPET)

## Data Availability

The data are contained within this article.

## Abbreviations

The following abbreviations were used in this manuscript:

PFO	Patent foramen ovale
R–L	Right-to-left
POS	Platypnoea–orthodeoxia syndrome
EID	Exercise-induced desaturation
RA	Right atrium
CPET	Cardiopulmonary exercise testing
ASD	Atrial septal defect
IVC	Inferior vena cava
SVC	Superior vena cava
LA	Left atrium
PED	Provoked exercise desaturation
SaO_2_	Arterial oxygen saturation
OSA	Obstructive sleep apnoea
TOE	Transoesophageal echocardiography
ODI	Oxygen desaturation index
AHI	Apnoea–hypopnoea index
CPAP	Continuous positive airway pressure
COPD	Chronic obstructive pulmonary disease
TTE	Transthoracic echocardiography
TCD	Transcranial Doppler
GOLD	Global Initiative for Chronic Obstructive Lung Disease
PaO_2_	Partial pressure of arterial oxygen
PAP	Pulmonary artery pressure
HAPO	High-altitude pulmonary oedema
EIAH	Exercise-induced arterial hypoxaemia
SpO_2_	Peripheral oxygen saturation
TIA	Transient ischemic attack
VO_2_max	Maximal oxygen uptake
PETCO_2_	End-tidal carbon dioxide pressure
VE/VCO_2_	Ventilatory equivalent for carbon dioxide
VE/VO_2_	Ventilatory equivalent for oxygen
PETO_2_	End-tidal oxygen pressure
FVC	Forced vital capacity
FEV1	Forced expiratory volume in 1 s
FEV**1**/FVC	Ratio of forced expiratory volume in 1 s to forced vital capacity
MEF 25–75	Mean expiratory flow between 25% and 75% of FVC
PEF	Peak expiratory flow.
VO_2_	Oxygen uptake
RER	Respiratory exchange ratio
SBF	Systemic blood flow
PBF	Pulmonary blood flow
Qp/Qs	Pulmonary-to-systemic flow ratio
